# Transcriptomic analysis of human norovirus NS1-2 protein highlights a multifunctional role in murine monocytes

**DOI:** 10.1186/s12864-016-3417-4

**Published:** 2017-01-05

**Authors:** Zabeen Lateef, Gregory Gimenez, Estelle S. Baker, Vernon K. Ward

**Affiliations:** 1 Department of Microbiology and Immunology, Otago School of Medical Sciences, University of Otago, 720 Cumberland St, Dunedin, 9054 New Zealand; 2 Otago Genomics and Bioinformatics Facility, University of Otago, Dunedin, 9054 New Zealand

**Keywords:** Human norovirus (HuNoV), NS1-2, Disordered protein, Murine norovirus (MNV), RNAseq, Transcriptomic analysis

## Abstract

**Background:**

The GII.4 Sydney 2012 strain of human norovirus (HuNoV) is a pandemic strain that is responsible for the majority of norovirus outbreaks in healthcare settings. The function of the non-structural (NS)1-2 protein from HuNoV is unknown.

**Results:**

*In silico* analysis of human norovirus NS1-2 protein showed that it shares features with the murine NS1-2 protein, including a disordered region, a transmembrane domain and H-box and NC sequence motifs. The proteins also contain caspase cleavage and phosphorylation sites, indicating that processing and phosphorylation may be a conserved feature of norovirus NS1-2 proteins. In this study, RNA transcripts of human and murine norovirus full-length and the disordered region of NS1-2 were transfected into monocytes, and next generation sequencing was used to analyse the transcriptomic profile of cells expressing virus proteins. The profiles were then compared to the transcriptomic profile of MNV-infected cells.

**Conclusions:**

RNAseq analysis showed that NS1-2 proteins from human and murine noroviruses affect multiple immune systems (chemokine, cytokine, and Toll-like receptor signaling) and intracellular pathways (NFκB, MAPK, PI3K-Akt signaling) in murine monocytes. Comparison to the transcriptomic profile of MNV-infected cells indicated the pathways that NS1-2 may affect during norovirus infection.

**Electronic supplementary material:**

The online version of this article (doi:10.1186/s12864-016-3417-4) contains supplementary material, which is available to authorized users.

## Background

Human noroviruses cause seasonal self-limiting gastroenteritis, with outbreaks common in densely populated centers such as hospitals, cruise ships, and rest homes. It is estimated that 18% of all acute gastroenteritis worldwide is caused by norovirus [1]. In the US, norovirus mortality rates are higher in individuals aged over 65 years, whereas hospitalization is more common in children under 5 years of age [2]. The majority of outbreaks in healthcare institutes are caused by the genogroup II.4 strains [3]. The current pandemic strain is Sydney 2012, which arose as a recombination event between New Orleans 2009, Apeldoorn 2008 and Osaka 2007 viruses [4]. The GII.4 Sydney strain (henceforth called HuNoV) is antigenically different to its parent strains showing low to nil blockade activity with monoclonal antibodies raised against previous pandemic norovirus strains [5]. Currently, there is no vaccine for norovirus and anti-viral treatments have yet to be developed [6].

Noroviruses are a group of positive-sense, single-stranded RNA viruses that are divided into 5 genogroups. The virus consists of three open reading frames (orfs), with orf1 encoding non-structural proteins essential for virus replication and orfs 2 and 3 encode viral proteins 1 and 2, respectively, that self-assemble into capsids. In addition, genogroup V, murine norovirus (MNV) has an extra open reading frame, termed orf 4 that encodes a virulence factor involved in apoptosis [7]. The orf1 encoding polyprotein is further cleaved into 6 proteins by the viral protease, to give rise to NS1-2 (p48), NS3 (NTPase), NS4 (p22), NS5 (VpG), NS6 (protease) and NS7 (RdRp) [8].

The NS1-2 protein is unique to noroviruses and contains a highly disordered proline-rich N-terminus [9], a putative C-terminal transmembrane domain, and caspase cleavage sites [10]. The protein sequence shows homology to the NlpC/P60 superfamily of circular permutated enzymes based on the presence of H-box and NC motifs predicted to form a catalytic domain. These permuted papain-like NlpC/P60 enzymes function as peptidases, amidases, and acyltransferases [11]. In addition, they are predicted to contain lipid-binding sites and play a role in the ubiquitin signaling pathway [12].

Cellular expression of NS1-2 protein shows a cytoplasmic distribution. The GI NS1-2 interacts with the vesicular protein SNARE [13] and causes Golgi disassembly [14]. The Golgi localization and disassembly was inhibited when the predicted hydrophobic transmembrane domain of NS1-2 was removed. The GIII bovine norovirus NS1-2 on the other hand does not co-localize with Golgi or ER markers [15]. During murine norovirus infection, NS1-2 shows cytoplasmic punctate distribution, with partial localization with ER and at the replication complex situated near the microtubule organizing center, implying that the protein may have additional roles in cellular manipulation beyond that of virus replication [16, 17]. There is very limited data on the role of norovirus NS1-2 protein during in vivo infection. In mice, MNV persistence and tropism for proximal colon was linked to the presence of a glutamate instead of aspartate at position 94 in the NS1-2 protein, indicating that the NS1-2 protein may play a role in maintenance of viral reservoirs [18].

It is unclear which cell type human norovirus infects during an in vivo infection, but in vitro studies showed that B cells can take up human norovirus in the presence of intestinal bacteria, with increased viral RNA in the supernatant over time, indicative of virus replication [19]. However, for the purposes of a transcriptomic study, the presence of bacterial RNA contaminants is not desirable. More recently, human norovirus replication has been shown in specialized enteroid cultures derived from intestinal stem cells in the presence of bile [20]. These stem cells are derived from patients undergoing intestinal biopsies for a variety of pathological conditions, which would complicate transcriptomic analysis. MNV grown in monocytes has been used a model system to study norovirus pathogenesis, including intracellular signaling and effects on immune-regulatory molecules [7, 21, 22]. Since murine monocytes are well characterised in terms of both differences and similarities to its human counterpart [23, 24], and MNV infections have been well studied [25, 26], an established murine monocyte cell line was used in this study.

The role of the HuNoV NS1-2 is unknown and this study uses transcriptomic analysis through RNA sequencing to identify the cellular pathways targeted by the HuNoV NS1-2 protein, compares it to the effects of MNV NS1-2 protein, and overall to MNV-infected cells using murine monocytes.

## Methods

### Cells

RAW-Blue™ cells (InvivoGen) were maintained in DMEM supplemented with 10% FCS and 200 ug/ml Zeocin™ (InvivoGen) at 37 °C with 5% CO_2_, and routinely passaged when 70–80% confluency was reached. Antibiotics were not added during MNV infection or RNA transfection experiments.

### Virus and RNA transcripts

MNV-1 (CW1-P3) was initially generated via reverse genetics [27], and purified as previously described [21]. MNV NS1-2 (nt 1–1028) and NS1-2 dis (nt 1–431) were cloned from full-length MNV-1 cDNA using forward primers containing a T7 promoter (underlined), the MNV 5′ UTR (lowercase), and nt 6–25 at the N-terminus of the MNV genome (5′-GAAATTAATACGACTCACTATAgtgaaATGAGGATGGCAACGCCATC-3′), and reverse primers containing stop codon (bold) and unique restriction enzyme sites (italics) (NS1-2 (nt 1024–1046): 5′-AGCAAGGTCGAAGGGTTATTCGGC-3′) and (NS1-2 dis (nt 409–431): 5′-GGTGGT*CTGCAG*TTACTCCAAGATAGAGCCGATCACAG-3′). The PCR products were confirmed for authenticity by sequencing. The Sydney GII.4 NS1-2 transcripts from 5′ to 3′ end, containing a T7 promoter, HuNoV 5′UTR, nucleotides 5–993 (NS1-2 full length) and nucleotides 5–409 (NS1-2 disordered) were obtained as synthetic genes from GenScript using the non-structural polyprotein sequence (Norovirus Hu/GII.4/Sydney/NSW0514/2012/AU GenBank AFV08794.1). Each gene had unique enzyme restriction sites flanking the sequences to allow for cloning into pUC8 vectors. The NS1-2 genes from both MNV and HuNoV were cloned into pUC8 vectors, linearized, then capped and poly(A)-tailed RNA transcripts were generated using the mMESSAGE mMACHINE T7 ultra transcription kit (Ambion). The RNA transcripts were purified, to remove unincorporated NTPs, enzymes, and buffer components, using the MEGAClear transcription clean-up kit (Ambion). Purified RNA from both HuNoV and MNV NS1-2 proteins were stored in 4 ug aliquots at −80 °C.

### Infection and transfection

MNV infection was performed in triplicate, at an MOI of 5 for 12 h as described previously [28]. Control cells were mock infected with media alone. NS1-2 and NS1-2 dis RNA for both MNV and HuNoV were transfected separately into murine monocytes using a Neon transfection system (Invitrogen); 4 ug of RNA was electroporated with 1 × 10^6^ cells using 1 pulse for 20 milliseconds at 1730 V. The electroporated cells were immediately placed into 2 ml of pre-warmed media (per well of 6-well plate) and incubated for 12 h. Control cells were mock transfected without the RNA. All transfections were performed in triplicate. The infected and transfected samples were collected for total RNA purification and the presence of virus protein expression was confirmed by western blot (MNV NS1-2 and NS1-2 dis) or mass spectrometry analysis (HuNoV NS1-2 and NS1-2 dis). Mass spectrometry was performed by the Centre for Protein Research, Dept. of Biochemistry, University of Otago (Dunedin, NZ). MNV infection was confirmed by western blot analysis using validated antibodies for NS1-2 and viral capsid proteins [9].

### RNA purification

The cell monolayer was washed with Dulbecco’s PBS (Sigma), and cells were lysed in 1 ml of TRIzol reagent (Invitrogen). RNA was extracted using the chloroform method; briefly, 200 ul chloroform was added to TRIzol samples, mixed vigorously, and incubated at RT for 2 mins. Samples were centrifuged and the RNA-containing top phase was collected (~350 ul). An equal volume of 70% ethanol was added before purifying RNA via the PureLink RNA mini kit (Invitrogen) as per manufacturers instructions. The RNA was eluted in RNase and endotoxin-free water, and stored at −80 °C. RNA quality was checked using a Bioanalyzer (Agilent Technologies) before transcriptomic analysis. Next generation sequencing service was provided by the Otago Genomics and Bioinformatics Facility using Illumina TruSeq™ RNA libraries on an Illumina HiSeq2000 platform, with 2 × 100 base pair paired end reads.

### Transcriptomic analysis

Sequencing reads were first trimmed for sequencing adaptor and then for quality at Phred score of Q20. Only paired end reads longer than 50 nucleotides were kept using the SolexaQA package [29]. Reads were mapped against the mouse genome version 10 using TopHat [30] and Bowtie 2 [31]. Read count were summarized at the transcript level using RefSeq annotation from the mapping using bed tools [32] and in-house PERL script. The differential expression analysis was performed using the EdgeR Bioconductor package [33]. The library size was corrected to take into account the sequencing depth [34]. Transcripts with less than 1 count per million (CPM) per replicate were removed. Read counts were then normalized using the trimmed mean of M-value (TMM) approach. Biological coefficient of variation was estimated using the triplicates. Differential expression (DE) analysis using a quantile adjusted conditional maximum likelihood (qCML) approach was conducted. The p-values were then adjusted using the Benjamini-Hochberg procedure with a threshold of 5% false discovery rate (FDR).

### Flow cytometry analysis

Raw-Blue cells were transfected or infected as indicated above and harvested at 12hpi into 70% ethanol. The fixed cells were permeabilised with PBS buffer containing 0.1% saponin and 0.1% bovine serum albumin. Cells were incubated with rabbit anti-TLR antibodies (TLR7 (Abcam 45371) and TLR8 (Abcam 180610); 1/50 dilution) for 20 min, washed and labeled with goat anti-rabbit Alexa488 antibody (Invitrogen) for a further 20 min. Labeled cells were washed thoroughly before being analysed using BD Fortessa. Each sample contained 20,000 gated cells for analysis using FlowJo 10.1.

### Alignment and phylogenetic analysis

Multiple sequence alignments were performed using the MUSCLE tool [35]. The alignment for phylogenetic analysis was edited to eliminate poorly aligned positions and divergent regions using Gblocks software [36]. Phylogenetic analysis was performed using MrBayes 3.2.3 [37] on the Phylogeny.fr web server [38] under WAG substitution model with 10,000 generations. The phylogenetic reconstruction was then visualized using FigTree 1.4.2 (http://tree.bio.ed.ac.uk/software/figtree). All polyprotein sequences were obtained from GenBank: GI.1 Norwalk 1968 AAB50465.1, GI.2 Southampton 1991 AAA92983.1, GI.6 Kingston 2010 AFH88382.1, GI.8 Nagoya 2008 AII73782.1, GI.9 AHA91653.1, GII AKE07105.1, GII.1 Hawaii 1971 AFS33557.1, GII.2 Malaysia 1978 AFX1658.1, GII.2 Taiwan AGT39205.1, GII.3 Korea 2006 ADK23786.1, GII.3 China 1978 AFX1655.1, GII.4 Japan 2011 BAU24947.1, GII.4 Osaka 2007 BAJ13911.1, GII.4 New Orleans 2010 AEX91909.1, GII.4 Sydney 2012 AFV08794.1, GII.6 Guangzhou 2011 AGC96534.1, GII.12 Taiwan 2010 AGT39196.1, GII.14 Saga 2008 ADE28700.1, GII.17 AKB94549.1, GII.17 ALG05459.1, GIII Jena AFQ00092.1, GIV NSW 2010 AFJ21375.1, GIV.2 feline AFD30969.1, GV MNV AEY83582.1, and GV rat AFV48050.1.

## Results and discussion

### Phylogeny and secondary structure of HuNoV NS1-2

**Fig. 1:**
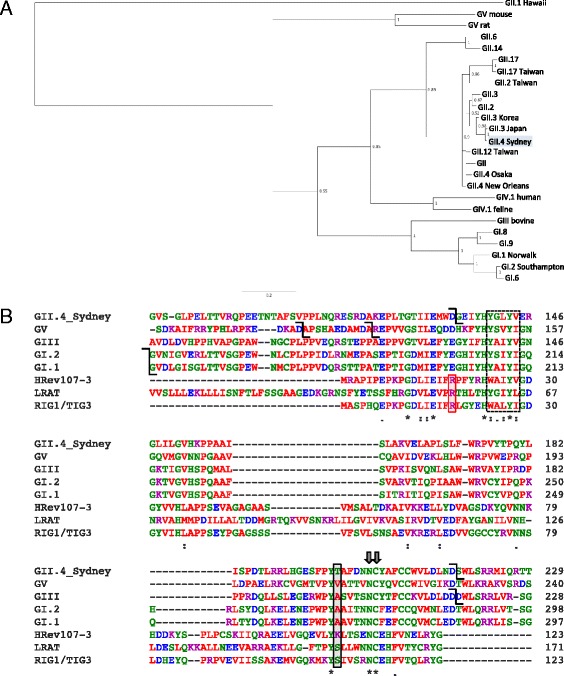
Comparison of NS1-2 proteins of norovirus genogroups. **a** Phylogenetic tree showing evolutionary distances between norovirus NS1-2 sequences. **b** Multiple sequence alignment of NS1-2 from the different genogroups with mammalian LRAT proteins, showing H-box (*dashed box*) and NC motifs (*double arrows*). The arginine (*red box*) required for mammalian phospholipase activity and tyrosine (*black box*) for acyltransferase activity is indicated. The predicted caspase cleavage sites are depicted by elbow connecters (*black*). Alignment generated using Clustal Omega; *denotes identity in all sequences, : indicates conserved substitutions, and . are semi-conserved amino acids

The sequences of the available norovirus orf1 proteins from Genbank were used to construct a phylogenetic tree showing the divergence of the NS1-2 proteins. The NS1-2 proteins cluster distinctly into their respective genogroups (Fig. 1a). The exceptions are the GII.1 Hawaii strain from 1971, which is different to the more current GII strains, and the GIII bovine strain clusters closely with the GI strains. There is significant variation between the GII strains, with the GII.4 Sydney (2012) NS1-2 protein clustering more closely with the GII.3 noroviruses than its parenteral GII.4 New Orleans strain consistent with the Sydney strain only sharing the orf2/3 capsid component with the New Orleans strain [4]. It is also interesting to note that the more recently emerging GII.17 and GII.14 strains are clustered separately to the GII.4 strains in terms of their NS1-2 identity (Fig. 1a). Alignment of NS1-2 proteins from norovirus genogroups shows that the HuNoV Sydney NS1-2 shares 42%, 36%, and 37% amino acid identity to the Norwalk (GI), Jena (GIII; bovine), and MNV (GV) NS1-2 proteins, respectively (Clustal Omega). Despite the low sequence homology, HuNoV NS1-2 shares certain traits with the other norovirus NS1-2 proteins. They all have a proline-rich disordered N-terminus (discussed in [9]) and a predicted transmembrane domain at the C-terminus based on concentration of hydrophobic residues [10]. The NS1-2 proteins also contain predicted caspase cleavage sites identified in GII.4 and GIII (DLxD*xWLS; probability 0.8), between the two GII.4 proteins (EMWD*GEIY; probability 0.7), and the two GI proteins (SARD*GVxx; probability 0.76) (Fig. 1b), as determined by Cascleave 2.0 [39]. The GV MNV NS1-2 has functional caspase-3 cleavage sites at residues 118 (DxxD*APSH) and 128 (DAMD*AKEP) [10]. The norovirus NS1-2 proteins also share the H-box and NC motifs (Fig. 1b) of the circular permutated NlpC/P60 family of peptidases [11]. Circular permutation allows proteins to adopt different enzymatic abilities within the same structure, and often leads to increased stability or reduced degradation by cellular proteases [40]. The proteins in the NlpC/P60 family are present in a wide array of prokaryotes and eukaryotic cells and function as peptidases, amidases, phospholipases, and acyltransferases. In the majority of the NlpC/P60 proteins, the catalytic domain is composed of a histidine (from H-box), a cysteine (from NC motif), and a third polar residue [12]. In bacterial NlpC/P60 cysteine peptidase enzymes, the most commonly present polar amino acid is a histidine, followed by asparagine, glutamate, glutamine, and aspartate [41]. In mammalian cells, the NlpC/P60 family includes LRAT (acyltransferase), HRev107-3, and TIG3 (class II tumor suppressors with phospholipase activity). Figure 1b shows the alignment of the conserved regions of norovirus NS1-2 proteins with mammalian NlpC/P60 proteins, highlighting the H-box and NC motifs and the additional polar residue. The polar residue determines substrate specificity; in HRev107-3, this residue is an arginine (Fig. 1b; red box) that is required to stabilize the phosphate group of phospholipid substrates [42]. In viral NS1-2 proteins, there is an acidic aspartate or glutamate instead of the basic arginine residue, suggesting that the HuNoV and MNV NS1-2 proteins may not function as a phospholipase or may utilize a different substrate. A viral NlpC/P60 protein, G6R from Vaccinia virus, also does not contain the arginine residue but was predicted to bind lipids based on the charge and hydrophobicity of the residues present in the catalytic binding groove [43]. The bacteria *Mycobacterium avium* subspecies *paratuberculosis*, which causes chronic intestinal disease in ruminants, contains a protein MAP_1204 belonging to the NlpC/P60 family that contains an acidic glutamate as the third polar residue, and this protein functions as a hydrolase [44]. The tyrosine residue upstream of NC motif in LRAT is required for its acyltransferase activity to generate all-trans-retinyl esters [12, 45], and this residue is conserved in the norovirus NS1-2 proteins. In addition, LRAT, HRev107-3 and RIG1/TIG3 have all been shown to induce apoptosis in cancer cells, and the presence of the NC motif is crucial for this function [46]. MNV-induced apoptosis has been attributed to the non-structural polyprotein, but has not been isolated to any individual protein in orf1 [47]. The structures of human LRAT and TIG proteins have been resolved, but due to circular permutation of the NlpC/P60 family, these mammalian proteins cannot be used to predict the structure of the norovirus NS1-2 proteins.

**Fig. 2:**
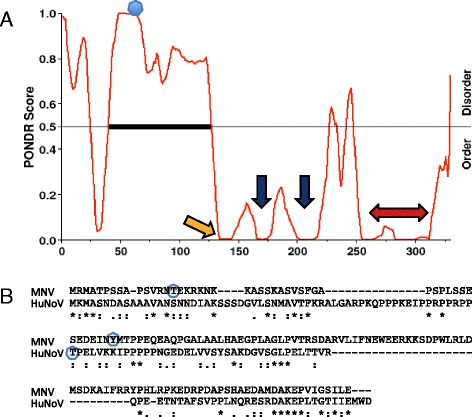
Disorder profile of GII.4 Sydney NS1-2 protein. **a** PONDR results showing predicted disordered region at the N-terminus of NS1-2 protein, the caspase cleavage site (*yellow arrow*; Cascleave), position of the H-box and NC motif (*blue arrows*), and the putative transmembrane domain (*red arrow*; PSIPRED) in the ordered region of the protein. The predicted phosphorylated threonine is indicated by a blue dot (DISPHOS). **b** Alignment of the disordered residues of MNV and HuNoV NS1-2 showing conserved motifs (Clustal Omega; * identical, : conserved substitutions, and . semi-conserved amino acids are indicated), and predicted phosphorylated threonine/tyrosine residues (*blue circles*; DISPHOS)

The analysis of HuNoV NS1-2 sequence using the Predictor of Natural Disordered Regions (PONDR^®^) [48] server showed 37% of the protein, consisting of amino acids 1 to 124 at the N-terminus, is disordered (Fig. 2a). This profile is similar to the previously published disorder profiles of GI.1, GI.2, GIII, and GV (MNV) NS1-2 proteins [9]. The predicted caspase cleavage site at amino acid 134 (EMWD-GEIY; 0.7 probability), at the termination of the inherently disordered region (yellow arrow; Fig. 2a) was chosen as the termination site for NS1-2 dis for this study. Figure 2a also shows the location of the H-box and NC motif (blue arrows), and the predicted transmembrane domain (red double arrow; PSIPRED) in the highly conserved/ordered region of the protein. The HuNoV NS1-2 secondary structure, hydropathy, and flexibility predictions are comparable to the previously published MNV NS1-2 protein analysis [9]. The disordered region of HuNoV NS1-2 contains limited secondary structure, and has residues that are predominately hydrophilic, flexible, and immunogenic, when compared to the full-length NS1-2 protein. Comparison of the inherently disordered regions shows areas of conservation between the HuNoV and MNV NS1-2 dis proteins that are not apparent with whole protein alignment (Fig. 2b). These similarities could play a role in interactions this disordered region has with other molecules. Analysis of MNV NS1-2 dis and HuNoV NS1-2 dis sequences using Prosite motif finder [49] showed that MNV NS1-2 dis protein did not match up to any known sequences, whereas the HuNoV NS1-2 dis showed sequence similarity to a putative *Mycobacterium* virulence protein predicted to bind to and inhibit IgG responses (NCBI-CDD 275319). The conserved motifs in MNV and HuNoV NS1-2 dis correspond to putative MHC-I epitope binding sites when compared against the Immune Epitope Database [50], indicating that the disordered part of NS1-2 may induce an immune response. Antibodies generated against full length MNV NS1-2 also bound the disordered N-terminal region of the protein, indicating that the disordered region is immunogenic [9], hence this disordered region may serve as a target for cell-mediated immune responses against norovirus.

The Disorder-Enhanced Phosphorylation Sites Predictor (DISPHOS 1.3) [51] predicted that the threonine-61 of HuNoV NS1-2 is phosphorylated, with a probability of 0.9 and 0.87, in human and murine cells, respectively (Fig. 2a and b; blue circle). Murine NS1-2 analysis using DISPHOS suggests that the protein may be phosphorylated at threonine-15 and tyrosine-47 (0.5 probability; Fig. 2b; blue circles). Threonine phosphorylation on kinase-type proteins are required for catalytic activity [52]. In eukaryotic cells, phosphorylation activates a variety of cellular pathways, including cell cycle regulation, apoptosis and immune cell activation [53]. RNA viruses, such as HIV and Hepatitis C, utilize host kinases to phosphorylate viral proteins in order to increase the viral proteome to aid virus replication [54, 55]. Hepatitis C virus NS5A protein contains a phosphorylated threonine amongst proline-rich disordered residues that is crucial for replication center formation and production of virions [56]. In MNV-infected monocytes, NS1-2 is partially localized to the replication complex but it is not known what role it plays in replication [16].

### HuNoV NS1-2 and NS1-2 dis induced transcriptomic profile

**Fig. 3:**
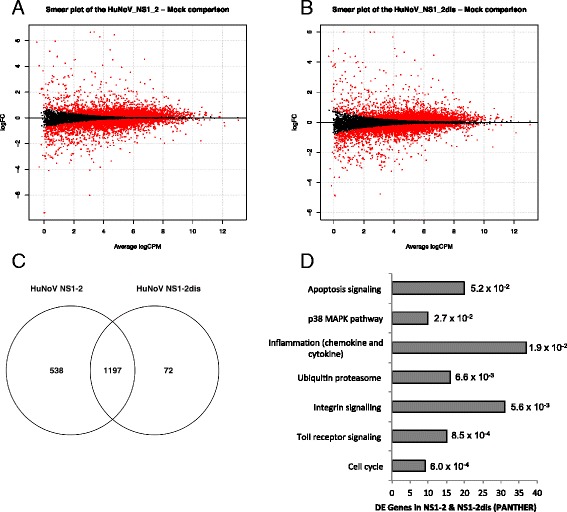
Genes differentially expressed in response to HuNoV NS1-2 and NS1-2 dis. Smearplots showing upregulated genes (*top panel*) and downregulated genes (*bottom panel*) in cells transfected with NS1-2 (**a**) and NS1-2 dis (**b**). **c** Venn diagram of the differentially expressed genes present in response to NS1-2 and NS1-2 dis proteins. **d** Genes present in both NS1-2 and NS1-2 dis were categorized into Panther pathways (y-axis) with the number of genes indicated (x-axis) using DAVID Bioinformatics Resources 6.7. The adjusted *p*-values (Benjamin-Hochberg) are indicated next to the bars

HuNoV NS1-2 and NS1-2 dis RNA transfections were carried out in triplicate, and cells electroporated without RNA were used as controls (mock-transfected). Cellular RNA was extracted and sequenced using Illumina TruSeq™ RNA libraries on an Illumina HiSeq2000 platform with 2 × 100 base pair paired end reads. The quality control, read mapping, read count, and differential expressed genes were performed as described in methods. The PCA plots of HuNoV NS1-2 and NS1-2 dis transfected cell samples showed that the triplicate samples clustered together and there is good separation between mock and viral protein transfected cells (Additional file 1: Figure S1). Smearplots of genes that are upregulated and downregulated in NS1-2 and NS1-2 dis are shown in Fig. 3a and b, respectively. Transfection of HuNoV NS1-2 altered the expression of 1735 genes, whereas HuNoV NS1-2 dis transfected cells had 1269 genes that changed compared to mock transfected samples (Cut off at 5% FDR, with 2-fold change). Of these differentially expressed (DE) genes, 1197 were shared between the two conditions (Fig. 3c). This left 538 DE genes found solely in the HuNoV NS1-2 transfected cells, suggesting that the ordered region of the viral protein in context with the disordered region was required for these cellular interactions. HuNoV NS1-2 dis transfected cells had 72 DE genes that were attributed solely to the disordered protein. The differentially expressed genes were further analysed by DAVID [57] and Panther [58] databases to categorise pathways and protein classes that were specifically up- or down-regulated in the presence of HuNoV NS1-2 and NS1-2 dis proteins.

Figure 3d shows the shared gene between the HuNoV NS1-2 and NS1-2 dis proteins classified into Panther pathways [59]. Using the Panther-db, majority of the DE genes are classified under inflammation mediated by chemokine and cytokine, integrin signalling, Toll-like receptor signalling, apoptosis, and ubiquitin proteasome pathways. The DE genes can also be classified separately into pathways that are either up- or down-regulated using Kegg classification. Table 1 shows that the upregulated genes belong to the pathways involved in proteasome, apoptosis, antigen processing and presentation, and gap junction interactions. The genes in RIG-I-like, NOD-like, and MAPK intracellular signalling pathways are also upregulated. The downregulated genes belong to the pathways involved in lysosomes, cell adhesion molecules, and transendothelial migration. In addition, the genes in p53, mTOR, and phosphatidylinositol signalling pathways are downregulated. There are some pathways where select genes are both up- and down-regulated, and these include cytokine-cytokine receptor interactions, Toll-like receptor signalling, focal adhesion, and regulation of actin cytoskeleton.

**Table 1:** DE gene pathways in HuNoV NS1-2 and NS1-2 dis transfected cells

	NS1-2 gene counts	NS1-2 dis gene counts	NS1-2 ^a^adjusted *p*-values	NS1-2 dis^a^adjusted *p*-values
Upregulated gene pathways				
Proteasome	25	15	1.80E^−15^	2.30E^−06^
Aminoacyl-tRNA biosynthesis	14	9	4.80E^−05^	2.30E^−02^
Focal adhesion	27	22	6.90E^−03^	2.60E^−02^
RIG-I-like receptor signaling	14	10	5.60E^−03^	6.20E^−02^
Toll-like receptor signaling	16	13	2.10E^−02^	5.30E^−02^
Pathways in cancer	33	28	7.80E^−02^	4.80E^−02^
NOD-like receptor signaling	11	8	7.20E^−02^	1.90E^−01^
Apoptosis	12	11	1.90E^−01^	9.50E^−02^
Regulation of actin cytoskeleton	22	16	1.80E^−01^	3.80E^−01^
Antigen processing and presentation	11	9	3.00E^−01^	3.50E^−01^
MAPK signaling	24	22	3.00E^−01^	1.50E^−01^
Gap junction	10	10	3.60E^−01^	1.90E^−01^
VEGF signaling	9	9	3.80E^−01^	1.90E^−01^
Cytokine-cytokine receptor interaction	-	20	-	1.80E^−01^
Downregulated gene pathways				
Lysosome	28	17	1.50E^−11^	4.30E^−05^
Leukocyte transendothelial migration	13	8	2.00E^−01^	6.30E^−01^
Phosphatidylinositol signaling system	9	9	3.30E^−01^	1.00E^−01^
Toll-like receptor signaling	10	7	3.70E^−01^	5.80E^−01^
Regulation of actin cytoskeleton	16	12	4.30E^−01^	7.30E^−01^
Glycosaminoglycan degradation	4	4	6.60E^−01^	7.50E^−01^
Cell adhesion molecules	11	-	7.10E^−01^	-
p53 signaling	-	6	-	8.50E^−01^
Cytokine-cytokine receptor interaction	-	13	-	6.80E^−01^
Focal adhesion	-	11	-	5.90E^−01^
mTOR signaling	-	5	-	5.40E^−01^

^a^adjusted *p*-values calculated using the Benjamini-Hochberg procedure

At first glance, these Kegg and Panther pathways appear to be diverse but they do contain similarities; the PI3-K and chemokine pathways require GTPases to function, and all the intracellular pathways use phosphorylation as a means of molecular activation [60, 61]. Further categorisation of these DE genes as molecular function (Kegg classification) showed that the types of proteins altered in response to HuNoV NS1-2 and NS1-2 dis can be classified as 43% phosphoproteins and 31% nucleotide-binding proteins, with 25% comprising solely of ATP-binding proteins (Table 2).

**Table 2:** Protein class of the DE genes in HuNoV NS1-2 and NS1-2 dis transfected cells

DE Genes NS1-2 and NS1-2 dis	% of gene counts	^a^Adjusted *p*-values
Phosphoprotein	43.2	7.6E^−02^
Purine nucleotide binding	31.1	6.6E^−06^
Nucleotide binding	31.1	5.5E^−06^
Ribonucleotide binding	28.8	1.5E^−05^
Purine ribonucleotide binding	28.8	1.5E^−05^
Adenyl nucleotide binding	27.3	7.3E^−06^
Purine nucleoside binding	27.3	6.0E^−06^
Nucleoside binding	27.3	5.3E^−06^
Cytoplasm	26.5	2.2E^−02^
ATP binding	25	2.1E^−05^
Transferase	18.9	5.8E^−04^
Hydrolase	16.7	2.0E^−02^

^a^adjusted *p*-values calculated using the Benjamini-Hochberg procedure

**Fig. 4:**
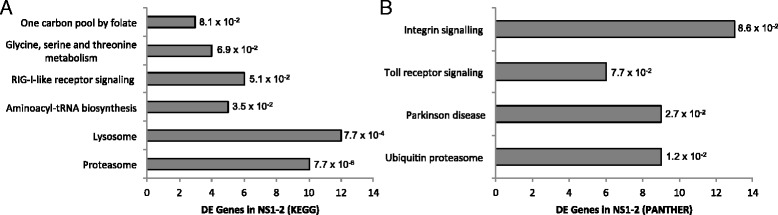
Genes differentially expressed in response to HuNoV NS1-2 only. Genes that were altered solely in response to transfected HuNoV NS1-2 were categorized into Kegg pathways (**a**) and Panther classification (**b**), with the number of genes indicated (x-axis) using DAVID Bioinformatics Resources 6.7. The adjusted *p*-values (Benjamin-Hochberg) are indicated next to the bars

The 538 DE genes present solely in HuNoV NS1-2 group mainly into the lysosome, proteasome, and RIG-1-like receptor signalling Kegg pathways (Fig. 4a). Panther classification of the same DE genes group to integrin-signalling, Toll-like receptors, and ubiquitin-proteasome pathways (Fig. 4b). When looking at the class of proteins that are altered by presence of HuNoV NS1-2, phosphoproteins made up 53% and acetylation constitutes 25% of the DE genes (data not shown). This suggests that the ordered region of the NS1-2 protein may play a role in regulating phosphorylated and/or acetylated proteins, as shown for mammalian proteins with the H-box and NC motif, such as LRAT and retinoic acid responder 3 (RARRES3) [62]. When looking at the cellular placement of the proteins altered by HuNoV NS1-2, 26% of the proteins are cytoplasmic and 29% are nuclear. Transfection of HuNoV NS1-2 into cells results in a diffuse cytoplasmic distribution with no nuclear localisation detected. Previous studies with Norwalk virus and MNV NS1-2 proteins have shown the same distribution pattern [13, 16]. The effects on nuclear proteins by HuNoV NS1-2 are more likely to be due to changes in transport across nuclear membrane that are related to modifications such as phosphorylation, rather than direct presence of NS1-2 in the nucleus.

The DE genes altered by the expression of HuNoV NS1-2 dis alone do not classify well into either Kegg or Panther pathways likely due to the small size of the gene set. Off the 77 DE genes, only 4 belong to the Ras pathway with GTPase function. The other DE genes do not correlate to any specific Kegg or Panther pathway, but do contain proteins that bind cations and/or specific motifs, such as zinc finger domains and kelch motifs (discussed below).

Taken together, the transcriptomic profile of murine cells expressing HuNoV NS1-2 and NS1-2 dis suggests that the virus protein affects multiple pathways. One of the possibilities based on *in silico* analysis is that HuNoV NS1-2 performs an enzymatic function. The enzymatic function of NS1-2 protein is likely to be related to phosphorylation, acetylation, or NTPase activity based on the pathways that are altered in cells expressing virus protein. The data also suggests that the disordered region of the NS1-2 protein may bind specific motifs or cations in substrate host protein that may lead to the enzymatic activity by the ordered region of the viral protein containing the H-box and NC motifs. As discussed previously, HuNoV NS1-2 contains the catalytic Y residue and NC motif in mammalian LRAT proteins required for acyltransferase activity and apoptosis, respectively.

### Comparison of HuNoV NS1-2 dis and MNV NS1-2 dis

**Fig. 5:**
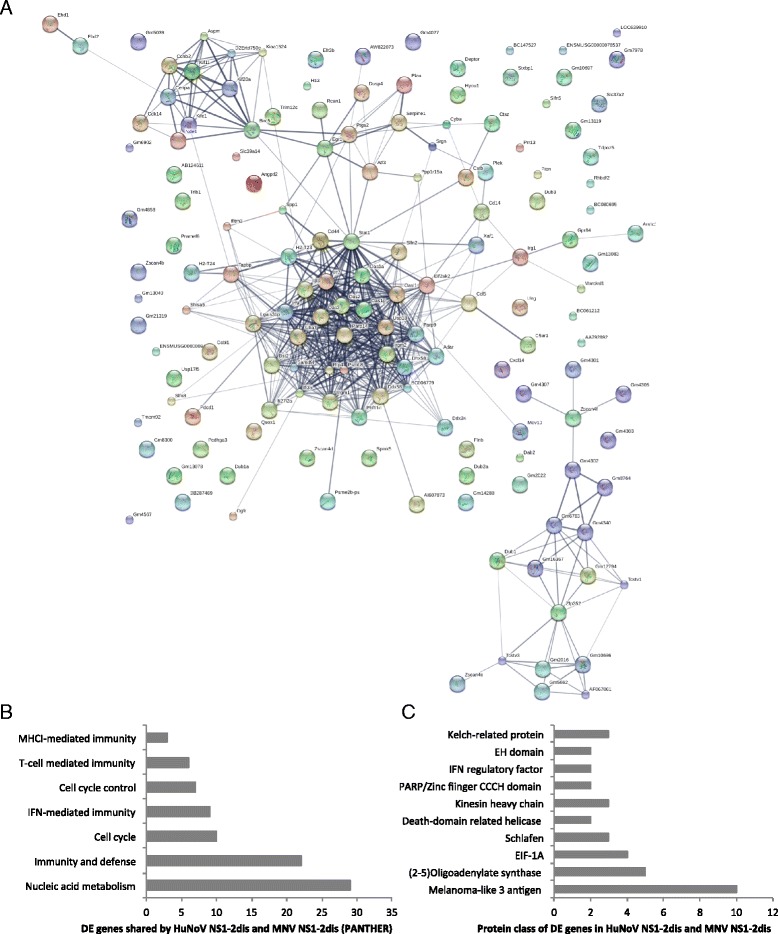
Genes in common between HuNoV NS1-2 dis and MNV NS1-2 dis. The DE genes present in both viral NS1-2 dis transfected cells were grouped using String-db (**a**). Genes were classified into biological process (**b**) and protein class (**c**) using Panther-db. The number of DE genes is shown on the x-axis with the pathway and protein classes indicated on the y-axis. Data generated using DAVID Bioinformatics Resources 6.7

Since the DE genes in HuNoV NS1-2 dis did not correlate well into distinct pathways, the changes in HuNoV NS1-2 dis transfected cells were compared to those in MNV NS1-2 dis cells. The disordered region of the HuNoV and MNV NS1-2 proteins share 153 DE genes when compared to mock-transfected cells. There is good correlation between the up- and down-regulated genes between the two viral proteins. Analysis of the up and downregulated DE genes present in both HuNoV NS1-2 dis and MNV NS1-2 dis using the String-db [63] showed presence of 2 distinct clusters involved in interferon regulation and cell cycle/apoptosis, with a further smaller cluster of genes with uncharacterised function (Fig. 5a). The upregulated genes of interest include the Schlafen gene family, *STAT1* and *IRF* transcription factors, indicating interferon-dependent activation in the NS1-2 dis protein transfected monocytes [64, 65]. Further, the genes induced by IFN upregulation, such as Oas enzymes, are also upregulated in both HuNoV and MNV NS1-2 dis samples, indicating that the NS1-2 dis protein activates a pro-inflammatory profile in monocytes. Analysis of the DE genes using Panther-db showed that they belong to the biological processes of nucleotide metabolism, cell cycle, IFN-mediated immunity, MHC-I, and T cells (Fig. 5b). The DE genes can also be grouped into protein families (Fig. 5c), with the highest number of genes in the melanoma-like 3 antigen family, the majority of which includes uncharacterised genes. Other protein families of interest, aside from the previously mentioned Schlafen and IFN proteins, include proteins with Kelch and EH domains, required for protein-protein interactions and intracellular sorting. The DE genes also include protein families with zinc finger motifs and helicases required for DNA binding (Fig. 5a and c). Overall, the number of immune response pathways triggered by the disordered region of NS1-2 from both HuNoV and MNV warrants further study in the context of virus infection.

### Comparison of HuNoV NS1-2 with MNV NS1-2 and correlation to MNV infection

**Fig. 6:**
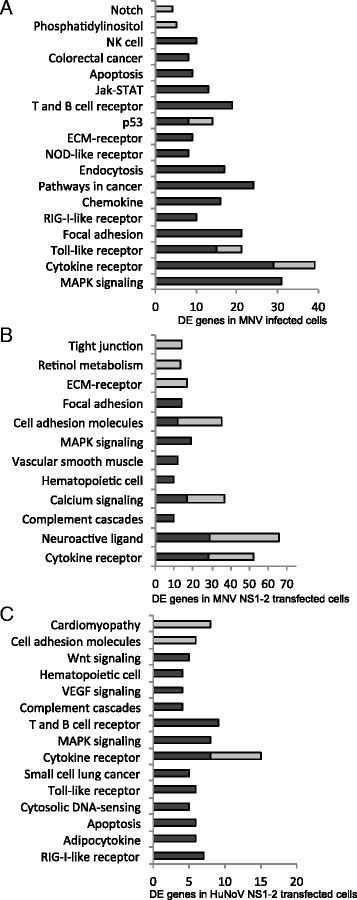
Comparison of DE genes in virus-infected and NS1-2-transfected cells. The stacked bars indicate DE genes that are either up- (*dark*) and down- (*light*) regulated genes in Kegg pathway classification (y-axis) in MNV-infected cells (**a**), MNV NS1-2 transfected cells (**b**), and HuNoV NS1-2 transfected cells (**c**). Data was analysed using DAVID Bioinformatics Resource 6.7

The HuNoV NS1-2 transcriptomic profile was compared to MNV NS1-2 transfected cells, and then overall to MNV infected cells. Figure 6 shows these up- and down-regulated genes classified into Kegg pathways. A closer look at the shared pathways shows changes in intracellular signalling molecules such as MAPK, and proteins that activate key immune responses, such as cytokines and chemokines. These DE genes and their fold changes are shown in Table 3. The genes present in MNV NS1-2 transfected cells followed similar regulation patterns to HuNoV NS1-2. In each case, the genes upregulated in HuNoV NS1-2 were also upregulated in MNV NS1-2 and consequently in MNV infected cells, showing a strong correlation between transfection of virus protein and whole virus infection in these specific pathways in monocytes.

**Table 3:** Select pathways in common between viral NS1-2 transfected cells and MNV infected cells

Gene	HuNoV NS1-2	MNV NS1-2	MNV infected
NFkB signalling			
Card11	−1.34	^a^NS	−0.88
Traf1	2.72	0.09	3.13
Ptgs2	2.01	0.19	2.29
Nfkb2	1.22	0.13	1.55
Relb	1.29	^a^NS	2.49
Ddx58	2.97	0.32	1.75
Tnfaip3	1.00	0.05	3.26
MAPK signaling			
Fgf13	−1.50	−0.10	−0.09
Myc	5.28	^a^NS	5.06
Mef2c	−1.27	−0.13	−0.22
Cacna1d	−3.04	−0.36	−0.86
Gadd45g	−1.21	−0.14	1.29
Dusp5	1.93	0.03	3.35
Dusp4	1.46	0.30	1.85
Dusp2	1.48	0.11	2.38
Nfatc1	1.36	0.12	0.70
Ptpn5	1.76	0.42	1.23
PI3K-Akt signaling			
Pkn3	1.20	0.25	0.54
Igf1	−2.58	−0.14	−0.30
Itgb5	−1.40	−0.03	−0.36
Lamc2	3.19	0.81	5.77
Creb3l2	1.29	0.28	1.23
Efna1	2.06	−0.13	−1.32
Pck2	1.08	0.19	0.45
Itga5	1.38	0.10	1.33
Lck	1.44	0.39	0.68
TLR signaling			
Ikbke	1.24	0.35	1.13
TLR8	−2.84	−0.20	−2.18
TLR9	−1.04	−0.04	−0.57
TLR4	−0.86	^a^NS	−0.47
TLR7	−0.88	^a^NS	−0.28
TLR13	−1.58	^a^NS	−0.68
IRF9	2.09	0.15	1.80
IRF7	4.51	^a^NS	2.88
IRF3	0.19	0.01	0.26
IRF1	−0.46	−0.1	−0.36
Apoptosis			
Fas	1.79	1.19	2.83
Capn2	1.07	0.04	0.21
IL1rap	1.11	0.08	1.12
IRAK2	1.19	0.18	1.97
Mlkl	1.56	0.32	0.29
Tnfrsf1b	−1.76	−0.07	−0.70
Metabolic pathways			
Fut7	−1.24	−0.14	−0.39
Ppt1	−1.06	−0.14	−0.07
Pla2g2d	1.53	0.18	0.73
Khk	−1.07	−0.23	−0.36
Ptgs1	−1.28	−0.07	−0.58
Pck2	1.08	0.19	0.45
Kmo	−4.09	−0.35	−1.64
Agpat4	1.46	0.11	1.34
Acy1	1.23	0.15	0.30
Il4i1	−1.67	−0.26	−0.03
Cth	2.61	0.54	1.91
Xdh	−2.46	^a^NS	−0.90
Acsbg1	1.76	0.08	0.63
St6gal1	−2.42	−0.08	−1.19
Dgkg	−2.86	−0.03	−0.68
Ugt1a7c	−1.22	−0.02	−0.22
Ak4	1.06	0.14	−0.27
Kdsr	−1.23	−0.15	−0.60
Acss2	−1.24	−0.02	−0.32
Mthfd2	1.19	0.04	0.59
Dhrs3	−1.90	^a^NS	−1.00
Dcxr	−1.02	−0.25	−0.36
Bdh2	1.96	0.32	0.98
Lpin3	1.22	−0.06	0.77
Gamt	−1.31	−0.06	−0.55
Akr1b8	1.45	0.15	0.69
Hpgds	−3.35	−0.04	−0.37
Alox5	−2.69	−0.06	−0.65
Chemokine and cytokine signaling			
CCL2	2.24	0.34	9.53
CCL3	1.01	0.03	2.22
CCL5	6.45	0.18	^b^ND
CXCL2	4.01	0.89	7.38
CCR1	2.53	0.50	1.20
CXCR3	1.09	0.11	0.09
CXCR4	−2.96	−0.14	−0.14
CX3CR1	−1.74	^a^NS	−0.43
TNF	1.85	0.11	2.54
Lif	2.28	0.52	3.48
Bcl3	1.25	0.40	1.54
Ifnar2	−1.24	−0.03	−0.55
Vegfa	1.60	0.09	0.79
Il10ra	−1.91	^a^NS	−1.43
Csf3r	−8.20	−0.27	−1.00

Positive logFC numbers indicate upregulation, and negative numbers are downregulated genes when compared to mock-treated and cells only samples

^a^ *NS*: gene detected, but FC not significant

^b^ *ND*: not detected

The combination of chemokine and cytokine mRNA that is upregulated in MNV infected cells suggests a Th1 profile, and this is further supported by down-regulation of genes such as IL10ra and CSF3R (G-CSF receptor) [66] that support an anti-inflammatory phenotype (Table 3). Overall, the transcriptomic profile suggests that MNV infected cells are pro-apoptotic, and affect MAPK, NFκB, and PI3K-Akt intracellular signalling pathways. The genes outlined in metabolic pathways affect the fatty acid and arachidonic acid regulation, with enzymatic activities such as oxidoreductase and transferase functions (Table 3). The proteins encoded by genes affected by MNV infection are localised at the endoplasmic reticulum and mitochondria. MNV NS1-2 expression induces a similar trend in intracellular signalling and metabolic pathways, apoptosis, and immune responses as MNV-infected cells, indicating that NS1-2 protein is a contributing factor to this phenotype, as these changes are not seen in mock-transfected or untreated monocytes. This same trend in all the pathways is seen with cells expressing HuNoV NS1-2 (Table 3), suggesting a similar role as MNV NS1-2 during virus infection. Previous work has shown that downregulation of survivin and upregulation of TNF and traf1 contributes to apoptosis during MNV infection [67]. The TNFα and Traf1 upregulation was also observed in this study, both in MNV infected and in NS1-2 transfected cells (Table 3). The chemokines secreted during an in vitro MNV infection correlate to a Th1 phenotype, and this has been previously shown by microarray analysis for mRNA and ELISA assays to detect secreted chemokines [21]. There is also an upregulation of serum TNFα and IFNβ [22], and an increase in inflammatory dendritic cells in the intestines of mice [68], during an MNV infection that further supports a Th1 phenotype.

**Fig. 7:**
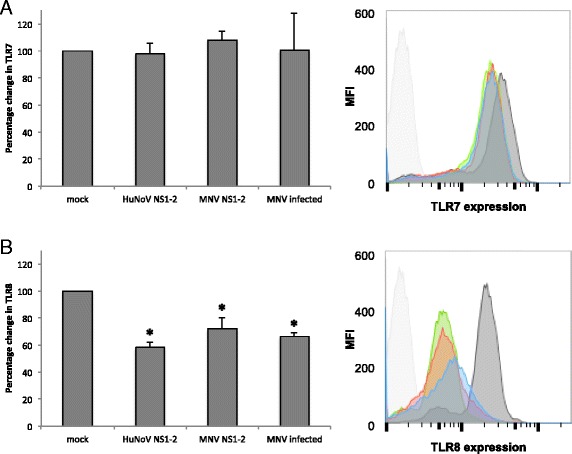
Toll-like receptor expression. Raw-Blue cells were infected with MNV or transfected with RNA constructs expressing HuNoV and or MNV NS1-2 for 12 h. Cells were stained with TLR7 and TLR8 antibodies and analysed using flow cytometry (BD Fortessa). The expression of TLR7 and TLR8 were normalized to mock transfected cells, and the percentage change in TLR expression is shown for **a** TLR7 and **b** TLR8. Data shown indicates average and standard deviation of 3 separate experiments. * *p* value <0.5, paired *t*-test. Representative flow cytometry plots from a single experiment with isotype control (*light grey*), cells control (*dark grey*), HuNoV NS1-2 (*blue*), MNV NS1-2 (*red*), and MNV infected (*green*) shown as histograms

The DE genes in the TLR pathway in this study indicate a moderate decrease in TLR4, TLR7, and TLR9 and a strong decrease in TLR8 mRNA in HuNoV NS1-2 transfected and MNV-infected cells. The changes in MNV NS1-2 transfected cells were not statistically significant (Table 3). The role of TLR regulation during norovirus infections is currently unknown, though the presence of viral RNA is known to upregulate intracellular TLR7/8 and TLR9 [69]. Previous studies have shown that there is good correlation between the mRNA and protein levels of TLRs, hence decreased mRNA levels indicate downregulation of the expressed TLR proteins [70]. To analyse this further, intracellular expression of TLR7 and TLR8 was analysed using flow cytometry in NS1-2-transfected cells and MNV-infected monocytes at the same time point as transcriptomic analysis. Figure 7a shows that expression of TLR7 does not change significantly in NS1-2 transfected or MNV-infected cells when compared to mock-transfected and untreated cells. However, expression of TLR8 (Fig. 7b) is decreased by 40% (HuNoV NS1-2), 35% (MNV NS1-2), and 37% (MNV infected) when compared to control cells. Interactions between TLR and single-stranded RNA viruses are not completely understood. Generally, TLR7/8 expression is increased upon recognition of viral single-stranded RNA, as shown for human parechovirus [71]. Some RNA viruses can modulate TLR expression either via direct binding to TLR or via modulating downstream effectors [69]. Hepatitis C virus encodes a non-structural disordered protein, NS5A which impairs TLR2 signalling by targeting MyD88 [69]. Studies with HIV-positive patients showed that the mRNA expression of TLR7/8 decreased during disease progression, and that activation of the TLR7/8 receptors led to suppression of HIV replication in monocytes [72]. In T cells however, HIV causes an upregulation of TLR7 resulting in T-cell anergy [73]. The consequences of down regulating TLR8 appear to be cell-specific. In mice, decreased TLR8 expression leads to an increase in TLR7 in dendritic cells but not in monocytes [74].

## Conclusion

This study undertook transcriptomic analysis of cells transfected with HuNoV NS1-2 to identify the cellular pathways affected by the virus protein. Our data indicates that the HuNoV NS1-2 protein targets multiple pathways in murine cells. The intracellular pathways include Jak-STAT, MAPK, p53, PI3K-Akt signaling, and the immune processes affected are apoptosis, chemokine and cytokine secretion, and TLR pathways. This phenotype suggests that HuNoV NS1-2 has a role in regulation of innate immunity, in particular affecting intracellular signaling pathways in response to cell surface receptor expression. The disordered region of HuNoV NS1-2 protein is predicted to be immunogenic and upon protein expression caused an upregulation of genes belonging to MHC-I, IFN, and T-cell mediated immunity pathways. Comparison of transfected HuNoV NS1-2 cells with MNV NS1-2 expressing cells showed a similar transcriptomic profile. Evaluation of the transcriptomic profile of MNV-infected monocytes correlated well with previous studies showing that MNV affects apoptosis and cytokine/chemokine secretion in monocytes. The RNAseq data indicates that NS1-2 protein may be responsible for these cellular immune functions. The role of TLR7/8 expression in MNV infection has not been previously reported, and our data shows that TLR8 expression in MNV infection is decreased at 12hpi, with no discernable change in TLR7 expression. The TLR7/8 profile is also present in NS1-2 transfected cells, showing a role for NS1-2 in modulating TLR signaling during infection. This study is the first to report the multiple pathways targeted by NS1-2 and identifies key areas that future research can focus on. In particular, the putative enzymatic function of NS1-2 that may be responsible for affecting multiple pathways needs to be determined.

## Additional file

Additional file 1: Figure S1. PCA plot showing triplicate samples of HuNoV NS1-2 or NS1-2 dis transfected cells compared to mock-transfected cells. (PDF 27 kb)

## Abbreviations

DE: Differentially expressed
ELISA: Enzyme-linked immunosorbent assay
HuNoV: Human norovirus
IFN: Interferon
KEGG: Kyoto encyclopedia of genes and genomes
LRAT: Lecithin retinol acyltransferase
MAPK: Mitogen-activated protein kinase
MHC: Major histocompatibility complex
MNV: Murine norovirus
NF-kB: Nuclear factor-kappa light chain chain enhancer of activated B cells
NS: Non-structural
PANTHER: Protein analysis through evolutionary relationships
TLR: Toll-like receptor
TNF: Tumor necrosis factor
