# Supplementary figures and images for: Transcriptomic analysis of human norovirus NS1-2 protein highlights a multifunctional role in murine monocytes
Source: BMC Genomics. 2017 Jan 5;18:39. doi: 10.1186/s12864-016-3417-4 (PMC5217272; doi:10.1186/s12864-016-3417-4)

PCA of 5.000 most variable genes

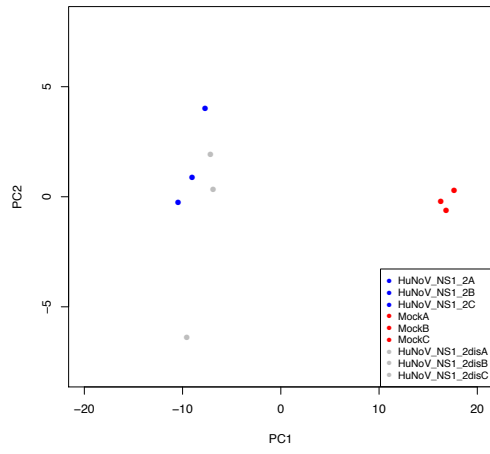

Supplement: Additional file 1: Figure S1. — PCA plot showing triplicate samples of HuNoV NS1-2 or NS1-2 dis transfected cells compared to mock-transfected cells. (PDF 27 kb) [file 12864_2016_3417_MOESM1_ESM.pdf]
